# Thermosetting Resins Based on Poly(Ethylene Glycol Fumarate) and Acrylic Acid: Rheological and Thermal Analysis

**DOI:** 10.3390/molecules30194020

**Published:** 2025-10-08

**Authors:** Gulsym Burkeyeva, Anna Kovaleva, Zhansaya Ibrayeva, David Havlicek, Yelena Minayeva, Aiman Omasheva, Elmira Zhakupbekova, Margarita Nurmaganbetova

**Affiliations:** 1 The Department of Organic Chemistry and Polymers, Chemistry Faculty, Karagandy University of the name of academician E.A. Buketov, Karaganda 100024, Kazakhstan; guls_b@mail.ru (G.B.); yelenaminayeva@yandex.ru (Y.M.); valihanovna@mail.ru (A.O.); elmira_zhakupbek@mail.ru (E.Z.); ritunur@mail.ru (M.N.); 2 The Department of Inorganic Chemistry, Faculty of Science, Charles University, Albertov 2030, 128 40 Prague, Czech Republic; david.havlicek@natur.cuni.cz

**Keywords:** unsaturated polyesters, acrylic acid, curing, DSC, kinetics, rheological behavior, thixotropy, TGA, thermostability, activation energy

## Abstract

The rheological behavior and low-temperature curing kinetics of poly(ethylene glycol fumarate)–acrylic acid systems initiated by benzoyl peroxide/N,N-dimethylaniline have been investigated for the first time with a focus on the development of thermosetting binders with controllable properties. It has been established that both composition and temperature have a significant effect on rheological behavior and kinetic parameters. Rheological studies revealed non-Newtonian flow behavior and thixotropic properties, while oscillatory tests demonstrated structural transformations during curing. Increasing the temperature was found to accelerate gelation, whereas a higher polyester content retarded the process, which is crucial for controlling the pot life of the reactive mixture. DSC analysis indicated that isothermal curing at 30–40 °C can be satisfactorily described by the Kamal autocatalytic model, whereas at 20 °C, at later stages, and at higher polyester contents, diffusion control becomes significant. The thermal behavior of cured systems was investigated using thermogravimetry. Calculations using the isoconversional Kissinger–Akahira–Sunose and Friedman methods confirmed an increase in the apparent activation energy for thermal decomposition, suggesting a stabilizing effect of poly(ethylene glycol fumarate) in the polymer structure. The studied systems are characterized by controllable kinetics, tunable viscosity, and high thermal stability, making them promising thermosetting binders for applications in composites, construction, paints and coatings, and adhesives.

## 1. Introduction

Thermosetting polymeric materials occupy a prominent position in the development of modern technologies due to their balanced combination of mechanical, thermal, and operational properties. Among them, thermosetting resins derived from unsaturated polyesters (UPs) are of particular interest, as they form robust and stable networks upon curing and are extensively employed in adhesives, coatings, composites, and electrical insulation [1,2,3,4,5]. A distinctive feature of these materials is the ability to undergo “cold” curing, i.e., the transition into a solid cross-linked state at room temperature without additional heating. Such systems not only reduce energy consumption but also enhance the reliability of the resulting materials, thereby underscoring the practical significance of research on unsaturated polyester resins (UPRs). Representative examples include polyesters synthesized from maleic anhydride or fumaric acid through polycondensation with various diols [6,7]. Depending on their molecular weight and degree of polymerization, these polyesters may exhibit either high or low viscosity.

Optimization of the synthesis and curing processes of UPRs is crucial for ensuring stable performance characteristics. The composition of monomers, the type of initiating systems, temperature, and the concentrations of catalysts and promoters exert a significant influence [8,9]. Effective optimization relies on the wide application of modern analytical techniques. Investigating the rheological properties of such systems is essential for evaluating processability, the ability to recover structure after mechanical deformation, and for optimizing processing conditions. The curing behavior of thermosetting resins is commonly analyzed using FTIR spectroscopy [10,11,12], rheometry [13,14], and differential scanning calorimetry (DSC) [15,16,17,18]. DSC enables a detailed examination of the entire curing process: the curing rate and degree of conversion as functions of time are determined from exothermic effects, which can be approximated by various kinetic models [18]. The most widely used approaches include the autocatalytic model proposed by Sourour and Kamal [15,18] and its modified version, incorporating diffusion control, developed by Khanna et al. [19,20].

A number of studies have focused on modeling curing kinetics, which makes it possible to establish a correlation between composition and kinetic parameters [21], and classical investigations of rheology and gelation, where the influence of catalyst concentration on the curing process has been demonstrated [9]. Recent works have revealed a relationship between the structure and thermomechanical characteristics of UPRs, which determine their performance properties [22]. Methods for evaluating gelation time and curing behavior in the presence of catalysts, promoters, and fillers [23], together with early studies on viscosity changes and gelation dynamics [24,25], are of particular importance. Additional research has addressed strategies for optimizing the processing of UPRs through the use of reactive diluents [26], as well as new approaches to resin functionalization, including the development of bio-oriented branched systems with enhanced thermal stability [27]. Issues of thermal stability and thermal degradation of the thermosetting polymer matrix have also been examined [28].

The potential of UPRs as innovative binders for the development of high-performance composite materials has been demonstrated [21,29]. However, most of these investigations have focused on conventional UPRs modified with styrene or methyl methacrylate [30,31,32,33], whereas the use of unsaturated carboxylic acids remains underexplored. Their incorporation provides opportunities for enhancing the functionality of UPR-based materials. In particular, acrylic acid (AA), due to its high reactivity, enables the regulation of crosslinking density and the improvement of the mechanical and performance properties of resins [34].

In the works [35,36], the kinetics of copolymerization of UPR systems with acrylic and methacrylic acids was investigated by dilatometry, and their physicochemical and thermal properties after curing at elevated temperatures were examined. In the study [37], the copolymerization kinetics of polypropylene glycol fumarate with AA was analyzed by DSC in the presence of a high-temperature initiator, and the thermal stability of the resulting materials was evaluated. However, to date, no studies have addressed the rheological properties, gelation time, structural transformations during curing, kinetic behavior based on DSC data, and thermal stability of UPs systems with unsaturated carboxylic acids cured under “cold” conditions in the presence of a redox-initiating system. In this study, poly(ethylene glycol fumarate) (p-EGF) was selected as the unsaturated polyester. The choice was determined by a combination of technological and structural factors. P-EGF exhibits high compatibility with AA. The presence of fumarate double bonds enables efficient radical copolymerization with AA, while the symmetric ethylene glycol chain without side methyl groups reduces steric hindrance and promotes the formation of a denser and more rigid network [38].

In this work, in order to optimize the conditions for obtaining a thermosetting binder based on p-EGF with AA that exhibits the required performance characteristics, the effects of composition and temperature on the rheological behavior and kinetic parameters of the curing reaction have been established. Particular attention has been devoted to the investigation of flow behavior, structural transformations during curing, and the thermal stability of the studied systems.

## 2. Results and Discussion

### 2.1. Curing of p-EGF-AA

**Figure 1:**
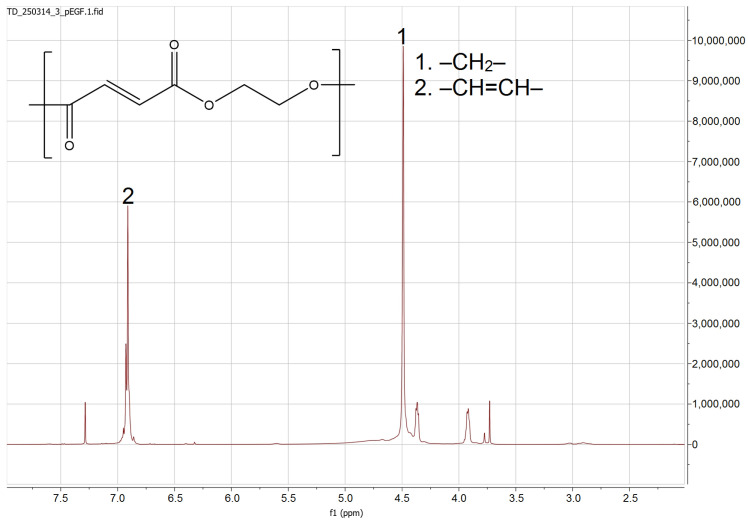
^1^H-NMR spectrum of p-EGF.

The precursor p-EGF was synthesized through a polycondensation reaction between ethylene glycol and fumaric acid [37]. The synthesized unsaturated polyester was identified using ^1^H and ^13^C-NMR spectroscopy (Figure 1). This method enabled the identification of the main structural elements of p-EGF. Characteristic chemical shifts corresponding to vinyl, methylene, and carbonyl groups were observed, confirming the structure of p-EGF. When interpreting the ^1^H-NMR spectra of p-EGF, it was taken into account that several groups of protons are present in the polymer (Figure 1). The first set of signals corresponds to the –CH=CH– moieties originating from the fumaric acid monomer, while the second refers to the –CH_2_– units derived from the ethylene glycol fragment. Vinyl protons of the –CH=CH– groups were detected in the range of 6.8–6.9 ppm as doublets. The ^1^H-NMR signals of the methylene protons –CH_2_– appeared as singlets in the region of 4.3–4.7 ppm.

**Figure 2:**
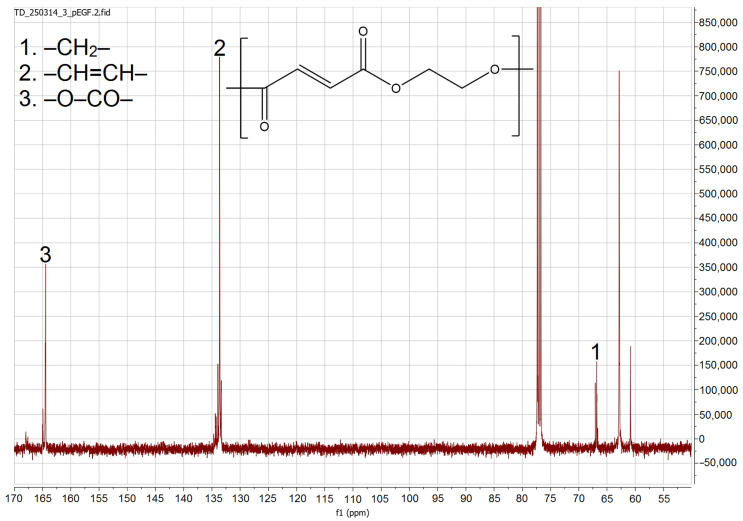
^13^C-NMR spectrum of p-EGF.

The presence of structural groups corresponding to fumarate and characteristic groups of ethylene glycol in the monomer was confirmed by the ^13^C-NMR spectra (Figure 2). In the ^13^C-NMR spectra, the carbon atoms of the methylene groups –CH_2_– from ethylene glycol resonate in the mid-field region at 60–70 ppm. Signals characteristic of the unsaturated –CH=CH– groups appear in the region of 132–135 ppm. The presence of signals at 163–165 ppm indicates the presence of carbon atoms associated with the ester group –O–CO–.

**Figure 3:**
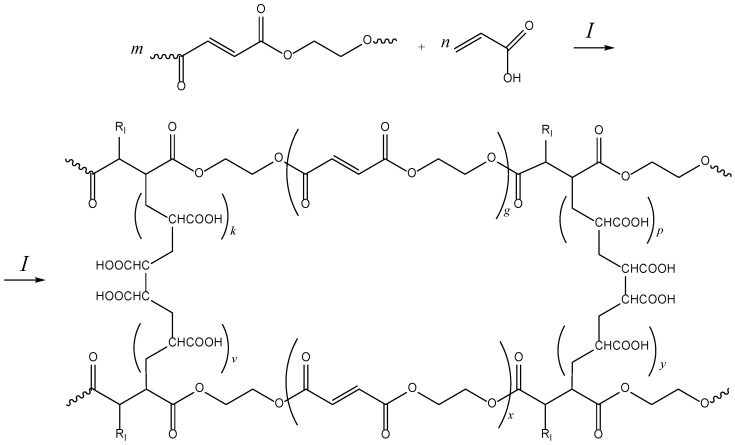
Synthesis of the copolymers of pEGF with AA. R_I_ is the radical of initiator.

Investigations into the curing reaction of UPs with styrene in the presence of two-component redox initiating systems have been reported in the literature [39]. However, similar work concerning the curing process of UPs with AA has not been described. A redox “cold” curing initiating system, comprising benzoyl peroxide (BPO) as the initiator and N,N-Dimethylaniline (DMA) as the promoter, was employed to study the curing behavior of p-EGF-AA-based reaction systems at relatively low temperatures. The scheme of the radical copolymerization of p-EGF and AA is presented in Figure 3.

### 2.2. Rheological Studies

#### 2.2.1. Flow Behavior of pEGF Solutions

The flow behavior of polymer solutions depends on various factors, including polymer concentration, molecular weight, and flow conditions. As is well known [40], polymer solutions may exhibit either Newtonian or non-Newtonian behavior. The study of the flow characteristics of polymer solutions is of great importance in industries such as adhesive and paint production, pharmaceuticals, and polymer processing, where control over flowability and viscosity is essential for ensuring the quality of the final product [41].

**Figure 4:**
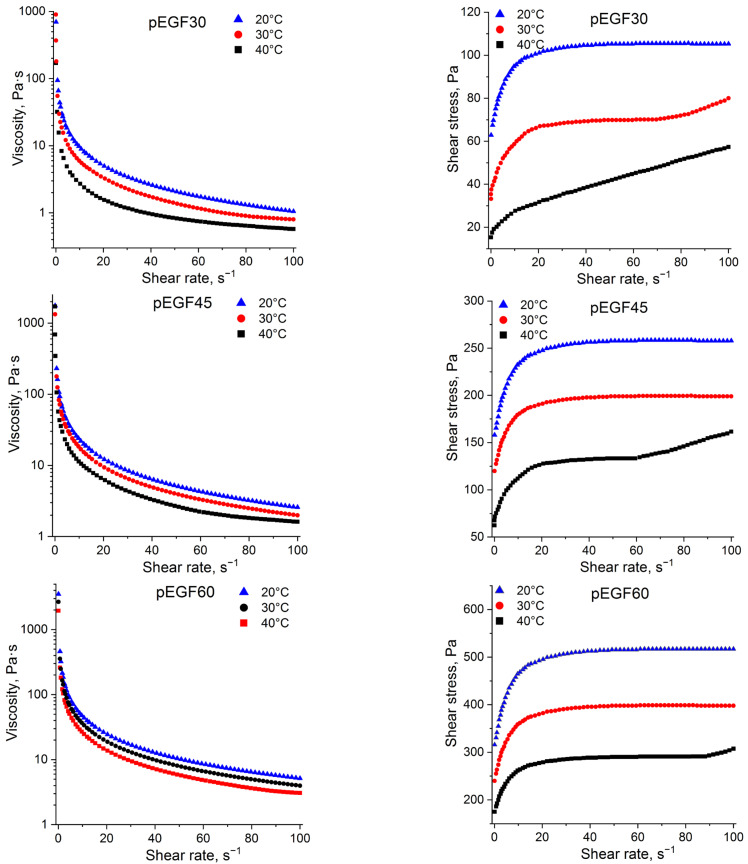
Graphs of apparent viscosity and shear stress dependences on shear rate for pEGF samples.

Figure 4 presents the dependencies of apparent viscosity and shear stress on shear rate for pEGF30, pEGF45 and pEGF60 solutions at 20 °C, 30 °C and 40 °C.

The conducted studies established that the apparent viscosity of the investigated pEGF systems decreases with increasing shear rate. This behavior is characteristic of non-Newtonian fluids, whose viscosity decreases with increasing shear rate, indicating shear-thinning behavior (i.e., at higher shear rates the material becomes less viscous). The flow characteristics of the studied solutions are influenced by both temperature [42] and the concentration of the unsaturated polyester. For instance, the pEGF30 system at 20 °C exhibits non-Newtonian flow behavior, with the shear stress becoming almost constant (Figure 4). At 30 °C, the flow curve acquires a linear character beginning at ~75 s^−1^, while at 40 °C this occurs already from ~10 s^−1^, indicating a transition of the pEGF30 solution to Newtonian behavior. For the pEGF45 and pEGF60 systems at 20 °C and 30 °C, an almost constant value of shear stress is observed with increasing shear rate, which indicates structural stability and non-Newtonian nature. At 40 °C, the shear stress begins to increase slightly with shear rate (~60 s^−1^ for pEGF45 and ~90 s^−1^ for pEGF60), which may suggest partial structural breakdown and a transition to Newtonian flow behavior [43].

#### 2.2.2. Investigation of Thixotropic Properties

Practical applications require that products such as paints, coatings, adhesives, construction materials, creams, ointments, and binders exhibit thixotropic properties. In a resting state, such products should possess sufficiently high initial viscosity, enabling them to retain their shape under mechanical stress. As is well known [44], thixotropic materials are characterized by a temporary reduction in viscosity under mechanical influence. Specifically, their flowability increases when subjected to stirring or applied force, while their viscosity recovers once the external force is removed [45]. Thixotropic testing enables the optimization of manufacturing processes, and allows for the prediction and improvement of the performance characteristics of final products [46].

In continuation of the rheological investigations, it was of interest to examine the thixotropic properties of the pEGF30, pEGF45 and pEGF60 systems. The thixotropic behavior was studied using an MCR 302e rheometer (Anton Paar). Based on the dependence of shear stress on shear rate, hysteresis loops were obtained, which made it possible to evaluate thixotropy. The hysteresis method proposed by Green and Weltman is used for the analysis and modeling of the behavior of materials with nonlinear mechanical properties, such as thixotropic substances. This method consists of systematically increasing and decreasing the shear rate.

**Figure 5:**
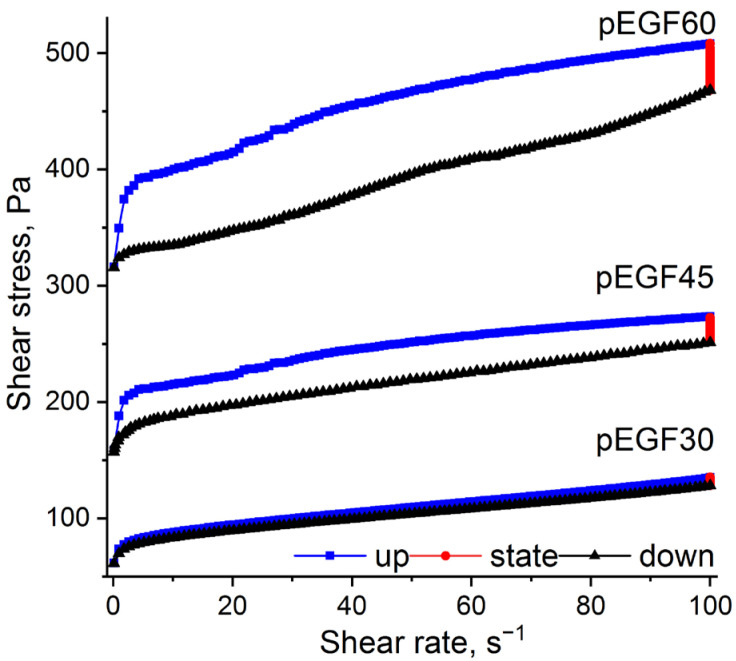
Thixotropic hysteresis loop of pEGF systems.

Graphically, the method is illustrated by closed loops in shear stress versus shear rate plots. Accordingly, a thixotropic material will exhibit a hysteresis loop due to the lag of shear stress behind shear rate [47]. The area enclosed by the hysteresis loop is used to characterize the degree of thixotropy. A larger loop area indicates more pronounced thixotropic properties of the solution. Figure 5 presents the shear stress versus shear rate curves for pEGF30, pEGF45 and pEGF60 solutions at 25 °C.

The presented rheograms show that the shear stress versus shear rate dependencies exhibit hysteresis loops, which are characteristic of thixotropic materials. It was found that the hysteresis loop areas for the pEGF45 and pEGF60 systems (2880.3 Pa/s and 6568.3 Pa/s, respectively) are significantly larger than that for pEGF30 (1553.5 Pa/s). This indicates more pronounced thixotropic behavior in the pEGF45 and pEGF60 systems compared to pEGF30. It was established that the content of unsaturated polyester influences the thixotropy of the investigated systems. An increase in polyester concentration leads to enhanced thixotropic behavior. This can be attributed to a higher proportion of long polymer chains, which are capable of forming temporary physical entanglements that increase viscosity under static conditions. Furthermore, increasing the concentration of unsaturated polyester results in a greater number of intermolecular interactions, which enhances resistance to flow in the absence of shear and manifests as thixotropic behavior.

#### 2.2.3. Rheological Analysis of Isothermal Curing

As a continuation of the rheological investigations, the curing process of the pEGF30, pEGF45 and pEGF60 systems was studied by rheometry at 20 °C, 30 °C, and 40 °C. As is well known [48], oscillatory testing is one of the most reliable methods for rheological characterization during polymerization. Taking into account the linear viscoelastic behavior,

(1) $$G^{*}=G^{\prime }+i\cdot G^{\prime \prime }$$

where $G^{\prime }$ is the storage modulus and $G^{\prime \prime }$ is the loss modulus.

The formation of a polymer network structure can be evaluated through rheological measurements by monitoring parameters such as the dynamic storage modulus, loss modulus, and viscosity. The storage modulus $G^{\prime }$ reflects the elastic component of the viscoelastic response, while the loss modulus $G^{\prime \prime }$ characterizes the viscous component, which can be regarded as the “liquid-like” behavior of the sample [49,50].

**Figure 6:**
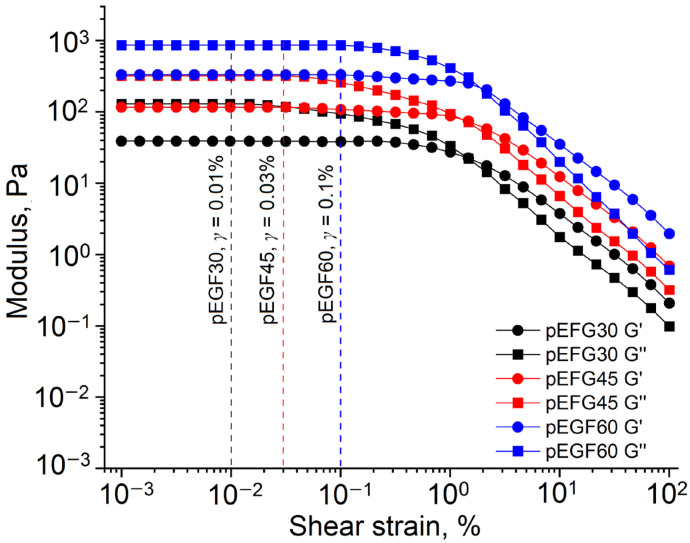
Strain sweep for pEGF systems at 40 °C.

Strain sweep tests (γ = 0.001–100%, ω = 10 rad/s) were performed at 20 °C, 30 °C, and 40 °C to determine the linear viscoelastic region (LVR) for each system (pEGF30, pEGF45, pEGF60). As an example, Figure 6 presents the sweep at 40 °C. The critical strain γ, at which $G^{\prime }$ deviates from the plateau, was determined using the 5% deviation criterion. Based on these results, a strain amplitude of γ = 0.01% was selected for all isothermal curing experiments, as it lies within the LVR for all pEGF systems and temperatures [51].

**Figure 7:**
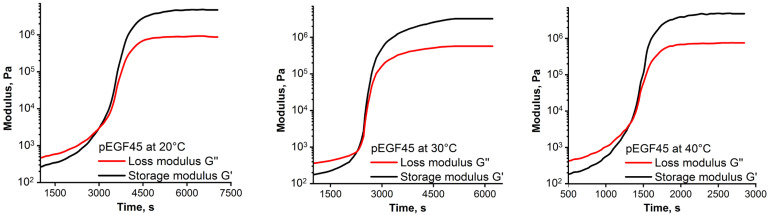
Time-dependent graphs of storage modulus and loss modulus during the isothermal curing process of pEGF45 system.

Figure 7 shows the time-dependent storage modulus ($G^{\prime }$) and loss modulus ($G^{\prime \prime }$) during the isothermal curing of the pEGF45 sample at 20 °C, 30 °C, and 40 °C. Analysis of the obtained rheograms allows three characteristic regions to be distinguished, reflecting the sequential stages of structural transformations of the investigated systems during curing. In the first region, G″ exceeds $G^{\prime }$ ($G^{\prime \prime }$ > $G^{\prime }$), indicating the dominance of viscous properties over elastic ones. This is associated with the low degree of polymer chain crosslinking at the initial stages of the reaction, when the samples are predominantly in a liquid state and the energy applied for deformation is mainly dissipated as heat. The second region corresponds to the gelation point, which is determined by the crossover of the $G^{\prime }$ and G″ ($G^{\prime }$ = $G^{\prime \prime }$). From a chemical perspective, this point marks the formation of an infinite three-dimensional network of covalently bonded macromolecules (i.e., the formation of a molecule with an infinitely large molecular weight). This determines the onset of elastic properties. In the third region, after the gelation point, the storage modulus $G^{\prime }$ exceeds the loss modulus $G^{\prime \prime }$ ($G^{\prime }$ > $G^{\prime \prime }$), indicating the predominance of elastic behavior over viscous behavior. This is related to the further progress of the curing reaction, an increase in crosslink density, and the formation of a more rigid three-dimensional network.

The obtained data (Figure 7) indicate a decrease in the gelation time (t_gel_) with increasing temperature. An increase in temperature enhances the mobility of active chains, leading to a faster curing reaction. The concentration of p-EGF also influences t_gel_. It was established that with an increase in the content of unsaturated polyester, the values of t_gel_ increase. For example, in the case of the pEGF30 sample, gel formation occurs at approximately 17 min at 40 °C, whereas at 20 °C it takes about 35 min (Figure S1). A similar pattern is observed for the pEGF45 (Figure 7) and pEGF60 (Figure S2) samples; however, the gelation times for these samples are longer and amount to ~26 min and ~33 min at 40 °C, and ~56 min and ~70 min at 20 °C, respectively. It is also noteworthy that the G′ values for pEGF45 and pEGF60 are higher than those for pEGF30, indicating a more elastic crosslinked structure in the pEGF45 and pEGF60 samples. The increase in $G^{\prime }$ from pEGF30 to pEGF60 indicates a rise in crosslink density as the p-EGF fraction increases. These results are consistent with the literature on UP-systems with unsaturated carboxylic acids, where higher UP content leads to a stiffer network and reduced chain mobility [35,36].

### 2.3. Isothermal and Dynamic DSC Results

The kinetic parameters of the p-EGF curing reaction were studied by DSC under isothermal and dynamic (post-isothermal) conditions [37,52].

**Figure 8:**
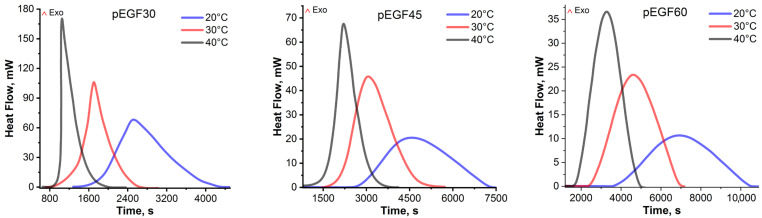
Isothermal DSC thermograms of the curing of pEGF samples.

Figure 8 presents the DSC thermograms of the isothermal curing of pEGF30, pEGF45 and pEGF60 samples at 20 °C, 30 °C, and 40 °C.

As seen in the thermograms (Figure 8), increasing the curing temperature results in more intense exothermic peaks that appear at shorter reaction times across all samples. The heat of curing is proportional to the degree of conversion (α), which can be expressed by the following equation:

(2) $$\alpha =\frac{\Delta H_{i}}{\Delta H_{tot}}$$

where $\Delta H_{i}$ is the reaction heat released at time t, (J/g); $\Delta H_{tot}$ is the total heat of the reaction, (J/g).

The total heat of curing ($\Delta H_{tot}$):

(3) $$\Delta H_{tot}=\Delta H_{i}+\Delta H_{r}$$

where $\Delta H_{r}$ is the residual heat released when the sample was post-cured in a dynamic DSC test in the temperature range of 20–200 °C after the first isothermal cure at a reaction rate of 10 °C/min.

Table 1 presents the enthalpy values of the curing reaction in the isothermal ($\Delta H_{i}$) and dynamic (post-isothermal, $\Delta H_{r}$) modes for the pEGF30, pEGF45, and pEGF60 systems at 20 °C, 30 °C, and 40 °C. The DSC experimental data show that with increasing temperature $\Delta H_{i}$ increases, while the residual heat $\Delta H_{r}$, recorded under dynamic conditions, decreases. For the pEGF30 sample, an increase in total heat release ($\Delta H_{tot}$) from 355.46 to 390.02 J/g is observed as the temperature rises from 20 to 40 °C, indicating a more complete curing process at elevated temperatures. During the curing of the pEGF45 and pEGF60 systems, a similar increase in $\Delta H_{tot}$ was observed—from 318.20 to 340.78 J/g and from 281.03 to 304.49 J/g, respectively—further confirming the acceleration of thermal reactions.

**Figure 9:**
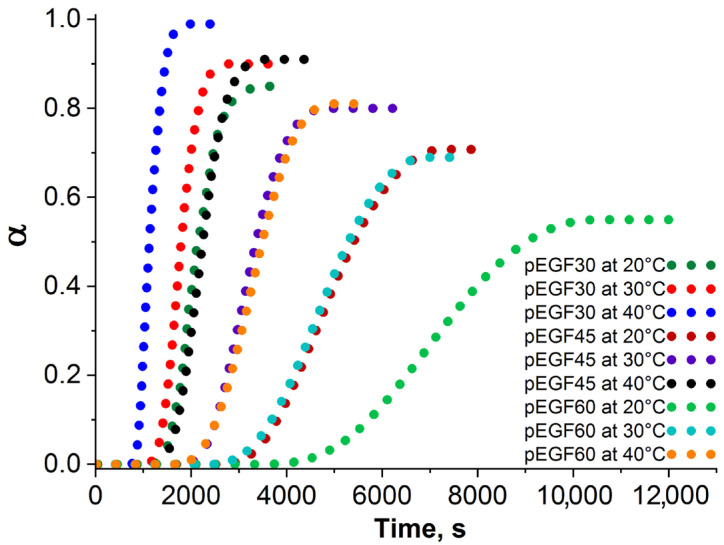
Conversion (α) as a function of curing time for the pEGF systems at different curing temperatures.

Figure 9 shows the conversion (α) versus curing time (t) curves for the pEGF systems at 20 °C, 30 °C, and 40 °C.

As seen in Figure 9, the conversion (α) increases rapidly at the beginning of the curing process. Subsequently, as the reaction proceeds, α stabilizes, indicating the completion of curing. With increasing temperature, the conversion α increases for all the investigated systems. For pEGF30, the α value increases from 0.85 at 20 °C to 0.99 at 40 °C; for pEGF45, from 0.70 to 0.91; and for pEGF60, from 0.55 to 0.81 at the same temperatures (Table 2). This indicates that at elevated temperatures the reaction reaches nearly complete conversion. The pEGF30 sample is characterized by a higher conversion degree α, suggesting a more intensive curing process. Increasing the temperature also reduces the curing time required to reach the maximum conversion values α. For the pEGF30 system, the conversion reaches α ≈ 0.85 at a curing temperature of 20 °C in ~2200 s. When the temperature is raised to 40 °C, the conversion increases to α ≈ 0.99 and is achieved within 1150 s. A similar pattern is observed for the pEGF45 and pEGF60 systems (Figure 9).

The isothermal curing process of the pEGF thermosetting systems can be described by the autocatalytic model proposed by Kamal [18,53,54]:

(4) $$\frac{d\alpha }{dt}=(k_{1}+k_{2}\alpha^{m})(1-{\alpha )}^{n}$$

where

α is the degree of curing;

m and n are the kinetic reaction orders;

$k_{1}$ the rate constant of the primary reaction step;

$k_{2}$ the rate constant of the autocatalytic process.

The effective rate constants $k_{1}$ and $k_{2}$ follow the Arrhenius law:

(5) $$k\left(T\right)=Ae^{\left(\frac{-E_{a}}{RT}\right)}$$

where

A is the pre-exponential factor;

$E_{a}$ is the activation energy;

R is the universal gas constant;

T is the absolute temperature.

In Equation (5), it is assumed that the activation energy $E_{a}$ and the pre-exponential factor A are independent of temperature within the investigated range. In this work, the Arrhenius analysis was performed for the parameter $k_{2}$, as its values are significantly higher than those of $k_{1}$. The activation energy $E_{a}$ was determined from the slope of the $lnk_{2}$ versus (1/T) dependence in accordance with Equation (5).

Table 2 presents the calculated values of the rate constants $k_{1}$ and $k_{2}$, as well as the reaction orders m and n for all pEGF systems at different temperatures. In all the systems studied, a consistent increase in both rate constants is observed with increasing curing temperature. It should be noted that in all cases k_2_ is greater than k_1_, indicating a stronger influence of k_2_ on the curing process [55]. The highest values of $k_{1}$ and $k_{2}$ (2.908·10^−4^ s^−1^ and 4.341·10^−3^ s^−1^) were obtained for the pEGF30 system at 40 °C, suggesting a high reactivity of this system. The ratio of the system components also affects the values of $k_{1}$ and $k_{2}$. In particular, the system with the highest content of unsaturated polyester, pEGF60, at the maximum temperature of 40 °C, exhibited the lowest values of $k_{1}$ and $k_{2}$ (1.601·10^−4^ s^−1^ and 0.909·10^−3^ s^−1^), which may be attributed to the reduced mobility of macromolecules resulting from higher viscosity.

**Figure 10:**
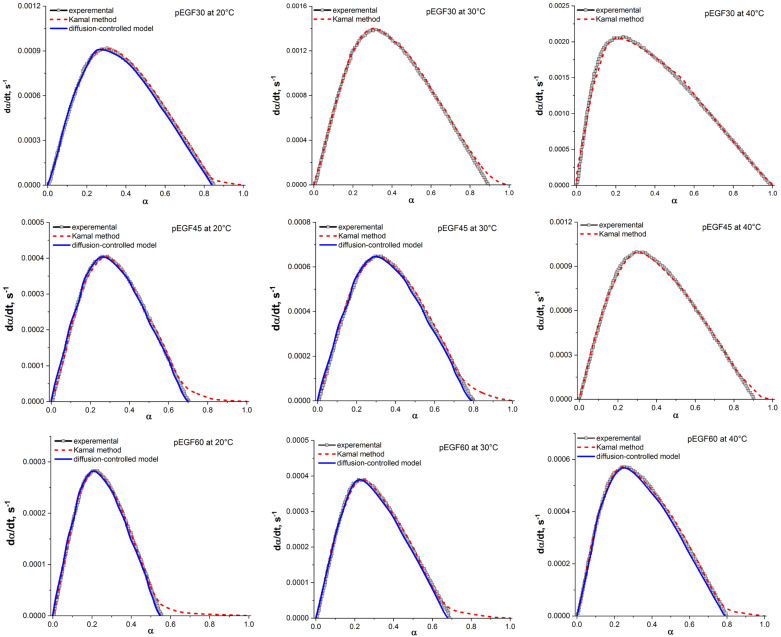
Plots of dα/dt versus α for the pEGF systems at different curing temperatures; gray lines—experimental data; red lines—fitted using the autocatalytic model; blue lines—fitted using the diffusion-controlled model.

The validity of the model was assessed by comparing its predictions with the experimental data. The curves predicted by the autocatalytic model, obtained using the parameters from Table 2, are presented in Figure 10.

The overall reaction order (m + n) of the investigated systems varies from 1.059 to 1.711 (Table 2), which is consistent with the autocatalytic curing mechanism characteristic of the radical polymerization of unsaturated polyester resins [56]. At the early stage of curing, chemical control predominates: active radicals interact with the double bonds of the monomers, leading to chain growth accompanied by heat release. The value of m + n decreases with increasing UP concentration in the systems, which is mainly attributed to a reduction in the reaction order n.

At the later stage of the process, the mobility of macroradicals becomes strongly restricted due to the increase in viscosity. As the curing reaction progresses, gelation and vitrification occur within the system, associated with an increase in crosslink density and polymer molecular weight [57,58]. This results in changes in the kinetic behavior of the systems, and at high degrees of conversion the reaction shifts to a diffusion-controlled regime. To account for the effect of diffusion control at the final stages of curing, a diffusion factor f(α) was introduced into Kamal’s autocatalytic model [18,59]:

(6) $$\frac{d\alpha }{dt}=(k_{1}+k_{2}\alpha^{m})(1-{\alpha )}^{n}\cdot f(\alpha )$$

where f(α) is expressed as

(7) $$f\left(\alpha \right)=\frac{1}{1+e^{C(\alpha -\alpha_{c})}}$$

Here, C is the modification constant, and $\alpha_{c}$ is the critical degree of curing determined by the glass transition temperature (T_g_) of the system. The value of f(α) can be obtained by dividing the experimentally determined reaction rate by the rate predicted by the Kamal model, while the parameters C and $\alpha_{c}$ are determined by fitting the data according to Equation (7).

When considering the isothermal curing of thermosetting resins, the introduction of the diffusion factor f(α) into the kinetic model is necessary to describe the later stages of the reaction, when chemical kinetics no longer determines the conversion rate. At this stage, a three-dimensional crosslinked structure (rigid network) is formed, in which the mobility of the reactants is significantly restricted, leading to noticeable deviations from the Kamal model.

The Kamal autocatalytic model shows good agreement with the experimental data for the overall curing process of the pEGF30 system at 30 °C and 40 °C, and for pEGF45 at 40 °C. However, at later curing stages and at lower temperatures, deviations are observed, indicating a diffusion-controlled process. For the pEGF30 system at 20 °C, deviations occur at $\alpha_{c}$ ≈ 0.83, while for pEGF45 deviations are observed at $\alpha_{c}$ ≈ 0.67 (20 °C) and $\alpha_{c}$ ≈ 0.78 (30 °C). Thus, with increasing temperature, the curing reaction deviates from the autocatalytic model at higher α values. For systems with a higher content of UP (pEGF60), deviations from the autocatalytic model are observed across the entire temperature range. With increasing temperature, the reaction order parameter n decreases (from 0.794 at 20 °C to 0.688 at 40 °C), indicating a reduced contribution of the autocatalytic mechanism to the overall kinetics. At lower temperatures, macroradicals are insufficiently mobile, and collisions between reactive groups are hindered; however, locally formed radicals retain high activity, which can temporarily accelerate the reaction. Deviations are observed at $\alpha_{c}$ values ranging from 0.50 to 0.79 as the temperature increases from 20 °C to 40 °C. Thus, the composition of the pEGF systems affects the overall curing kinetics.

As shown in Table 2, the value of α_c_ is slightly lower than α_max_ at the corresponding temperatures. As the temperature increases, α_c_ also increases. At the initial stage of the curing reaction, $\alpha_{max}$ < $\alpha_{c}$, f(α) → 1. As the curing reaction proceeds, the process shifts from chemical control to diffusion control upon reaching α_c_. At later stages of the reaction, i.e., when α >> $\alpha_{c}$, f(α) ≈ 0, and the reaction terminates. The approximated curves (blue line in Figure 10), obtained using Equation (7) and the parameters from Table 2, show good agreement with the experimental results.

The thermal effect, degree of conversion α, and curing rate of the pEGF30, pEGF45 and pEGF60 systems are also influenced by the concentration of the initial reactants, namely the unsaturated polyester and the carboxylic acid. This is evidenced by the apparent activation energy ($E_{a}$) values, which are 32.24 kJ/mol for the pEGF30 system, 35.89 kJ/mol for pEGF45, and 40.01 kJ/mol for pEGF60. These results suggest a lower reactivity of p-EGF compared to acrylic acid, leading to a reduction in curing time and copolymerization rate with increasing unsaturated polyester concentration [37,60].

**Figure 11:**
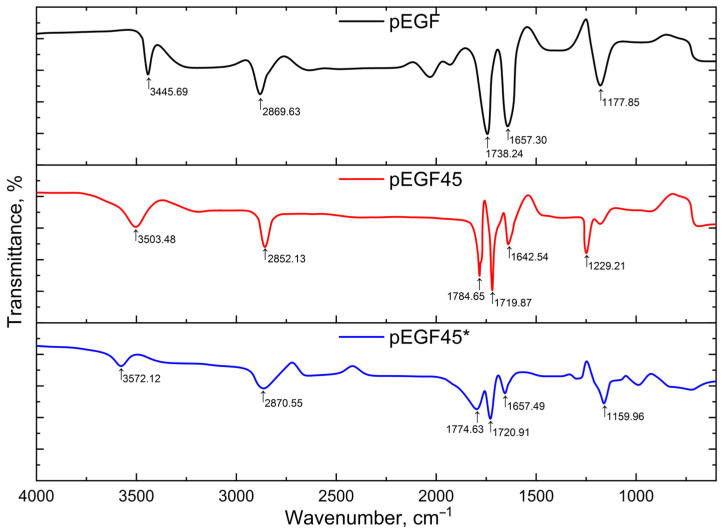
IR spectra of the initial p-EGF and its copolymers with AA: pEGF45—sample after isothermal curing at 20 °C; pEGF45*—sample after dynamic (post-curing) conditions.

The characterization of the cured samples was carried out using IR spectroscopy. Figure 11 presents the IR spectra of the initial p-EGF and its copolymers with AA at different degrees of curing: pEGF45 represents the sample subjected to isothermal curing at 20 °C, while pEGF45* refers to the sample obtained via dynamic (post-curing) conditions.

The presence of absorption bands in the region of 1640–1660 cm^−1^ in the IR spectrum corresponds to the unsaturated double bonds of the polyester moiety –C=C–. Strong, sharp bands observed in the range of 1100–1250 cm^−1^ indicate the presence of –CH_2_–CO– groups. The ester group –COOC=C– is identified by a characteristic peak at approximately 1738 cm^−1^.

The curing reaction of the initial p-EGF with AA involves the cleavage of double bonds (–C=C–) and the formation of a three-dimensional polymer network. The presence of peaks at 1642 cm^−1^ and 1657 cm^−1^ in the IR spectra of pEGF45 and pEGF45* indicates that a certain number of unreacted –C=C– bonds of the polyester remain. The decrease in the area of these peaks during the curing process is attributed to an increased number of addition reactions between acrylic acid units and p-EGF through the double bonds. The presence of carboxylic acid groups (–COOH) in the IR spectra of the copolymers pEGF45 and pEGF45* is identified by peaks at 1719 cm^−1^ and 1784 cm^−1^, and at 1720 cm^−1^ and 1774 cm^−1^, respectively. The methylene groups (–CH_2_–) from AA are observed at 2852 cm^−1^ and 2870 cm^−1^. The intensity of these peaks increases as a result of dynamic curing (post-curing), which is attributed to the higher content of AA segments in the molecular structure of the final product [61].

### 2.4. Thermogravimetric Analysis (TGA)

**Figure 12:**
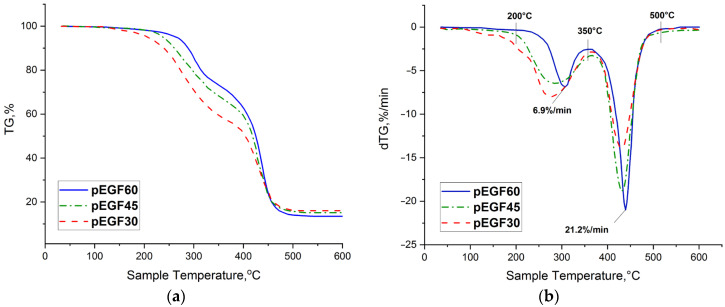
TG (**a**) and dTG (**b**) curves of the cured pEGF samples.

To investigate the thermostability of the cured pEGF30, pEGF45 and pEGF60 samples, thermogravimetric analysis (TGA) was employed. Figure 12 shows the mass loss of the samples isothermally cured at 40 °C as a function of temperature in the range of 30–600 °C. All samples exhibit a two-stage thermal degradation pattern, reflecting the complex nature of chemical transformations associated with the presence of both polyester and polycarboxylic fragments.

At the initial stage of degradation, cleavage of side chains and weakly bound functional groups occurs. In the system with a lower p-EGF content, the first degradation stage takes place in the range of approximately 120~360 °C, accompanied by a mass loss of about 40%. The primary processes include thermal decomposition of the polyacrylate phase: decomposition of β-carboxyl radicals, intramolecular dehydration of carboxyl groups with water elimination, and depolymerization of unstable chains [62,63]. Within this temperature interval, the disruption of weak hydrogen bonds formed between hydroxyl and carboxyl groups during copolymerization is also possible. With an increase in the p-EGF content to 45 mol.%, the onset of the first degradation stage shifts to a higher temperature—around 190 °C—indicating enhanced thermal stability due to the presence of polyester units in the structure. In the pEGF60 sample, degradation starts at 205 °C and continues up to 350 °C with a mass loss of approximately 23%. This behavior is attributed to the higher thermal resistance of pEGF fragments compared to acrylate segments.

At the second stage of decomposition (350~500 °C), all samples exhibit degradation of the main polyester backbone of p-EGF. The thermal breakdown is likely accompanied by scission of ester bonds and cleavage of macromolecular chains, resulting in the formation of low-molecular-weight compounds such as aldehydes, ketones, and hydrocarbon fragments [64]. The formation of cyclic anhydrides and cross-linked structures is also possible, partially stabilizing the polymer matrix.

Based on the results of the thermogravimetric analysis, further investigation was preferably conducted using the pEGF45 sample. The thermostability of the samples cured at different temperatures was subsequently examined, with the corresponding data presented in Table 3.

As shown in Table 3, increasing the curing temperature leads to a shift in the thermal decomposition intervals toward higher temperature ranges. At a curing temperature of 40 °C, a more complete reaction of the fumarate groups with the curing agent occurs, resulting in the formation of a denser and more uniform three-dimensional polymer network, which requires a greater amount of energy to break the chemical bonds. More complete polymerization reduces the amount of residual reactive monomers that decompose at lower temperatures. Higher curing temperatures may also promote the formation of a greater number of crosslinks within the polymer structure. At the same time, a comparison of the pEGF45 samples isothermally cured at 20 °C, 30 °C, and 40 °C shows that the thermal stability metrics are practically indistinguishable. This indicates that network formation is sufficiently complete in all cases. It should be noted that, according to the TGA data, additional post-curing occurs at the initial stage of heating (prior to the onset of thermal decomposition), which promotes full curing and increases the degree of conversion. The results are consistent with the DSC data obtained in both isothermal and dynamic (post-isothermal) regimes, as well as with studies [37] reporting that the crosslinking reaction is essentially completed at temperatures ≤100 °C for unsaturated polyester systems with unsaturated carboxylic acids.

**Figure 13:**
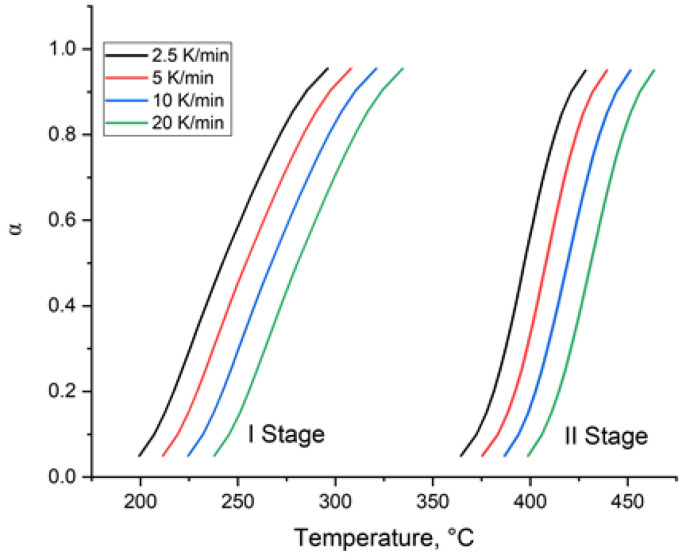
Temperature dependence of the conversion degree (α) during thermal decomposition.

To gain a comprehensive understanding of the decomposition process, the kinetics of thermal degradation of the pEGF45 sample cured isothermally at 40 °C was investigated. For the calculation of kinetic parameters, data obtained at different heating rates (2.5, 5, 10, and 20 °C/min) were used. Figure 13 presents the conversion degrees (α) of pEGF45 at the two distinct stages of thermal decomposition.

**Figure 14:**
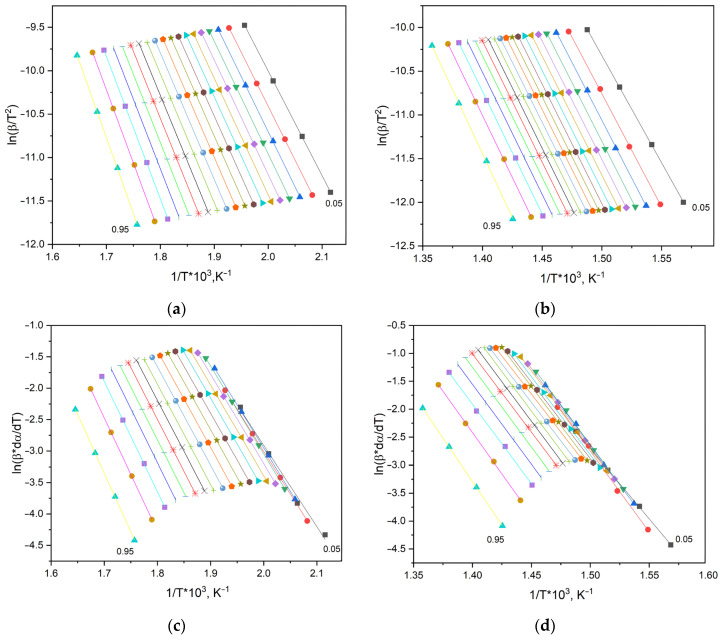
Graphical representations of the Kissinger–Akahira–Sunose and Friedman equations for the pEGF45 sample: (**a**,**b**)—stage I of decomposition; (**c**,**d**)—stage II.

As shown in Figure 13, the conversion curves calculated at different heating rates are positioned nearly parallel to each other, confirming the high quality of the experimental TGA data. The calculation of effective activation energy parameters was performed using the isoconversional methods of Kissinger–Akahira–Sunose and Friedman [65]. The linearized plots of the data are presented in Figure 14.

As shown in Figure 14, the data points lie almost on a straight line, indicating that the thermal degradation of the polymer follows a single kinetic mechanism within the considered range of conversion degrees (α).

**Figure 15:**
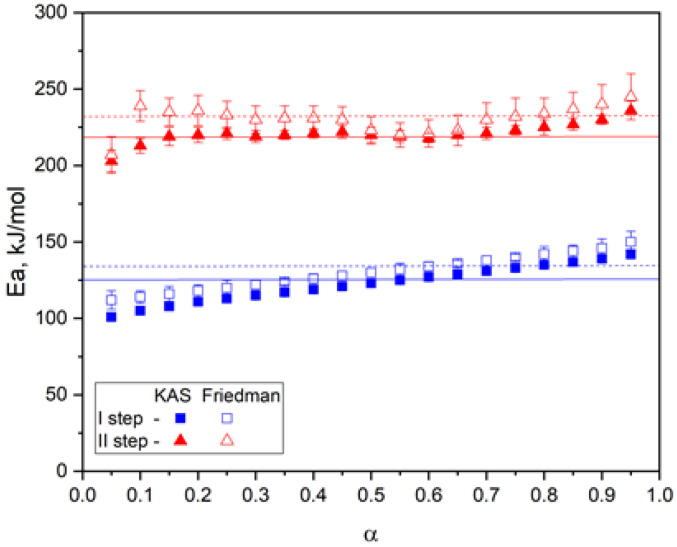
Dependence of activation energy (E_a_) on the degree of conversion (α) for the two decomposition stages of pEGF45.

Figure 15 shows the dependence of activation energy (E_a_) on the degree of conversion (α) for the two stages of thermal decomposition of the cured pEGF45 sample.

The E_a_ values in Figure 15 exhibit characteristic changes as a function of the conversion degree α, reflecting the complexity of the degradation processes. The activation energies obtained using both methods show good agreement, confirming the reliability of the derived kinetic parameters. The first stage of decomposition is characterized by low E_a_ values at the initial stage (α ˂ 0.2), which may be attributed to the cleavage of the weakest bonds or thermally unstable components, such as residual monomers. In the range 0.3 ≤ α ≤ 0.7 E_a_ reaches its maximum and remains relatively stable, indicating the predominance of one or more primary degradation processes. The average activation energy values for the first stage, as calculated using the Kissinger–Akahira–Sunose (KAS) and Friedman methods, were 120 ± 1.2 kJ/mol and 129 ± 2.8 kJ/mol, respectively. For the second stage of decomposition, the values obtained by the integral and differential methods are nearly twice as high (E_KAS_ = 227 ± 1.9, E_FR_ = 234 ± 2.8). The high E_a_ values during the second stage indicate that the degradation process is associated with the cleavage of strong covalent bonds within the main polymer chain.

## 3. Materials and Methods

### 3.1. Materials

The following reagents were used in this work: fumaric acid and acrylic acid (AA) (“Vekton”, Saint-Petersburg, Russia); ethylene glycol (99.8%) (“Ekos-1”, Moscow, Russia); zinc chloride catalyst (“Reachem”, Moscow, Russia); benzoyl peroxide (BPO), Luperox A75 (“Sigma-Aldrich”, Burlington, MA, USA); and N,N-Dimethylaniline (DMA) 99.5% (“Chemical line”, Saint-Petersburg, Russia) as a promoter.

### 3.2. Preparation of p-EGF

The initial p-EGF was synthesized through a polycondensation reaction between fumaric acid and ethylene glycol, following a procedure similar to that described in previous studies [37].

### 3.3. Curing of p-EGF

The curing of the p-EGF-AA reaction mixture was carried out by radical bulk copolymerization at various molar ratios of comonomers at temperatures of 20 °C, 30 °C, and 40 °C, in the presence of a redox initiating system [37,66], consisting of BPO as the initiator and DMA as the promoter. The concentrations of BPO and DMA were 1% and 0.15%, respectively, relative to the mass of the initial pEGF-AA mixture. The designations and formulations of the investigated samples are presented in Table 4.

### 3.4. Methods

#### 3.4.1. NMR Analysis

NMR spectra were recorded using a Bruker 600 MHz Avance III spectrometer (VTT). Deuterated chloroform (CDCl_3_) was used as the solvent. The measurements were carried out at room temperature.

#### 3.4.2. Gel-Permeation Chromatography

The molecular weight of p-EGF was determined by gel permeation chromatography (GPC) using a Malvern chromatograph equipped with a dual-detector system (Viscotek 270). According to the results, the weight average molecular weight (Mw) of the polymer was found to be 3620 Da [37].

#### 3.4.3. Rheological Analysis

Rheological studies of the pEGF samples were carried out using an MCR 302e rheometer (Anton Paar, Graz, Austria). In the rotational mode, the flow behavior and thixotropic properties of the pEGF30, pEGF45, and pEGF60 samples were investigated (CP40-1 geometry; cone diameter 40 mm, cone angle 1°, gap 0.08 mm) at 20 °C, 30 °C, and 40 °C, prior to the onset of the curing process. These tests made it possible to obtain flow curves for pEGF30, pEGF45 and pEGF60 along with the corresponding dependence of apparent viscosity on shear rate in the range of 0.1 to 100 s^−1^. The thixotropic properties were investigated using the thixotropic loop test (hysteresis loop method) at 25 °C. The hysteresis loop measurements were carried out as follows: the shear rate was first linearly increased from 0.1 to 100 s^−1^ over a period of 300 s, then held constant at 100 s^−1^ for 180 s, and subsequently decreased linearly back to 0.1 s^−1^ over another 300 s.

Oscillatory tests to determine the viscoelastic properties (storage modulus G′, and loss modulus G″) of pEGF30, pEGF45, and pEGF60 during curing were performed using a PP-25 plate system (25 mm diameter, 1 mm gap). Measurements were conducted isothermally at 20, 30, and 40 °C within the linear viscoelastic region (LVR) at ω = 10 rad/s and γ = 0.01%. The LVR boundaries were determined from strain sweep tests (a logarithmic strain range from 0.001 to 100%) at 10 rad/s.

Temperature control of the system was maintained using a convection temperature chamber equipped with Peltier elements. Both the rheometer and the temperature control system were operated using RheoCompass™ software from Anton Paar.

#### 3.4.4. Differential Scanning Calorimetry (DSC)

The kinetics of radical copolymerization (curing) of the pEGF30, pEGF45 and pEGF60 systems in the presence of BPO initiator and DMA promoter, as well as the residual reactivity of the samples, were studied using DSC with a Labsys Evolution TG-DTA/DSC simultaneous thermal analysis instrument (Setaram, Caluire-et-Cuire, France). A reaction mixture weighing 95–100 mg was placed in open alumina crucibles and loaded into the calorimeter measurement cell. The tests were carried out under isothermal conditions at temperatures of 20 °C, 30 °C, and 40 °C in an inert atmosphere [37].

After completion of the isothermal stage, the calorimeter cell was rapidly cooled to room temperature. Upon reaching thermal equilibrium, dynamic heating was performed from 20 °C to 200 °C at a heating rate of 10 °C/min to determine the residual heat of the curing reaction [37]. The curing reaction was considered complete when the exothermic curve leveled off to the baseline, indicating the absence of further heat release.

Each experiment was conducted three times. The kinetic parameters were calculated based on the resulting data and are presented as mean values with standard deviations, ensuring the reliability and reproducibility of the results.

#### 3.4.5. Thermogravimetric Analysis (TGA)

The investigation of the thermal properties of the pEGF30, pEGF45 and pEGF60 systems was carried out using a Labsys Evolution TG-DTA/DSC simultaneous thermal analysis instrument (Setaram, France) under dynamic conditions within a temperature range of 30–600 °C. The samples were heated in Al_2_O_3_ crucibles at heating rates of 2.5, 5, 10, and 20 °C/min in a nitrogen atmosphere, with a nitrogen flow rate of 30 mL/min. The instrument was calibrated for thermogravimetric measurements and heat flow analysis using CaCO_3_ and indium (In) standards, respectively.

The kinetics of thermal degradation is typically expressed by the following equation:

(8) $$\frac{d\alpha }{dt}=k\left(T\right)f(\alpha ),$$

where dH(t)/dt is the rate of heat flow;

k(T) is the reaction rate constant.

Formally, the temperature dependence of the rate constant can be expressed by the Arrhenius Equation (5).

It should be noted that the activation energy is the only parameter that allows for a direct comparison of the kinetic characteristics of the process. The combination of Equations (5) and (8) yields

(9) $$\frac{d\alpha }{dt}=A{exp}^{-\frac{Ea}{RT}}\cdot f(\alpha ),$$

The obtained equation provides the basis for differential kinetic methods. Since it is not possible to solve the right-hand side of Equation (9) analytically, various approximate methods are employed in practice.

The Friedman differential method was derived by applying the iso-conversional approach to Equation (9), resulting in [67]

(10) $${\ln\left(\frac{\mathrm{d\alpha}}{\mathrm{dt}}\right)}_{\alpha ,\;i}=ln\left[f\left(\alpha \right)A_{\alpha }\right]-\frac{E_{a}}{RT_{\alpha ,i}},$$

The index i is introduced to designate different temperature programs. $T_{\alpha ,i}$ represents the temperature at which a given conversion (α) is reached under temperature program i. As a result, Equation (10) assumes that $T_{\alpha ,i}$ changes linearly with time, with a heating rate of $\beta_{i}$.

There are several integral iso-conversional methods, which differ in their approximation of the temperature integral in Equation (10). The approximation of Murrey and White leads to B = 2 and C = 1, resulting in another well-known equation, often referred to as the Kissinger–Akahira–Sunose equation [68]:

(11) $$\ln\left(\frac{\beta_{i}}{T_{\alpha ,i}^{2}}\right)\;=Const-\frac{E_{a}}{RT_{\alpha ,i}},$$

The activation energy for the different conversion degrees is calculated from the slope of the dependence of $\ln\left(\frac{\beta_{i}}{T_{\alpha ,i}^{2}}\right)$ versus 1/T.

#### 3.4.6. IR-Spectroscopy

The cured products pEGF30, pEGF45 and pEGF60 were identified by IR spectroscopy. The IR spectra were recorded using KBr pellets on an FSM 1201 spectrometer [61].

## 4. Conclusions

To optimize the conditions for obtaining a thermosetting binder with the required performance characteristics, the rheological behavior and low-temperature curing kinetics of p-EGF with AA in the presence of the BPO/DMA initiating system were investigated for the first time. It was established that the system composition affects both the rheological behavior and curing kinetics. The systems exhibit non-Newtonian flow with thixotropic characteristics; moreover, increasing the UP content enlarges the hysteresis loop area from 1553.5 Pa/s (pEGF30) to 2880.3 Pa/s (pEGF45) and 6568.3 Pa/s (pEGF60), enabling viscosity control during processing. The formation of a cross-linked network during isothermal curing of the pEGF systems proceeds in three stages: predominance of viscous behavior (G″ > G′), gelation point (G′ = G″), and growth of elastic properties (G′ > G″). It was found that increasing the temperature accelerates gelation, whereas higher polyester content delays it, which is important for controlling the pot life of the reactive mixture. Higher G′ values observed for pEGF45 and pEGF60 indicate the formation of a more rigid structure.

DSC analysis demonstrated that the isothermal curing kinetics of the systems are satisfactorily described by the Kamal autocatalytic model at 30 °C and 40 °C. At 20 °C and at the later stages of curing, deviations were observed, which are associated with the reduced mobility of macroradicals and the onset of diffusion control. For pEGF60, discrepancies with the autocatalytic model were recorded over the entire temperature range. It was determined that an increase in polyester content raises the activation energy E_a_ of curing from 32.24 kJ/mol (pEGF30) to 40.01 kJ/mol (pEGF60).

According to TGA data, an increase in the proportion of p-EGF is accompanied by enhanced thermal stability. Activation energy E_a_ analysis as a function of conversion (α) confirmed the multistage character of thermal degradation (pEGF45, 40 °C): at the first stage, E_a_ was 120 kJ/mol (KAS) and 129 kJ/mol (Friedman), while at the second stage it reached 227–234 kJ/mol, which indicates the cleavage of strong covalent bonds. Thus, pEGF-based systems can be considered effective thermosetting binders. The use of such systems ensures controllable curing and high thermal resistance of materials, making them promising for applications in composite, construction, coating, and adhesive materials.

## Figures and Tables

**Table 1:** Reaction heats obtained from isothermal and dynamic DSC curve.

Sample	Temperature, °C	$-\Delta H_{i}$, J/g	$-\Delta H_{r}$, J/g	$-\Delta H_{tot}$, J/g
pEGF30	20	302.56 ± 6.96	52.90 ± 1.22	355.46 ± 7.21
	30	340.44 ± 7.49	36.83 ± 0.74	371.27 ± 8.12
	40	386.12 ± 8.11	3.90 ± 0.08	390.02 ± 8.09
pEGF45	20	222.60 ± 5.12	95.56 ± 2.10	318.20 ± 7.21
	30	264.18 ± 5.55	66.78 ± 1.34	330.96 ± 6.76
	40	310.85 ± 6.22	29.93 ± 0.60	340.78 ± 6.82
pEGF60	20	155.80 ± 3.90	130.23 ± 2.61	281.03 ± 6.41
	30	199.92 ± 4.80	90.99 ± 1.82	290.91 ± 6.62
	40	245.96 ± 5.17	58.53 ± 1.17	304.49 ± 6.33

**Table 2:** Kinetic parameters of the curing reaction of pEGF samples obtained from DSC.

Sample	Temperature, °C	$\alpha_{max}$ _max_	$k_{1}$·10^−6^, s^−1^	$k_{2}$·10^−3^, s^−1^	m	n	m +n	C	$\alpha_{c}$	R^2^	$E_{a}$, kJ/mol
pEGF30	20	0.85	2.003	1.863	0.599	1.112	1.711	24.7	0.83	0.9969	32.24
	30	0.90	2.446	2.880	0.628	1.067	1.695	-	-	-	
	40	0.99	2.908	4.341	0.669	1.008	1.677	-	-	-	
pEGF45	20	0.70	1.407	0.789	0.469	0.953	1.422	22.3	0.67	0.9908	35.89
	30	0.80	1.899	1.301	0.500	0.891	1.391	25.7	0.78	0.9959	
	40	0.91	2.301	2.022	0.533	0.828	1.361	-	-	-	
pEGF60	20	0.55	0.821	0.299	0.277	0.794	1.071	20.9	0.50	0.9926	40.01
	30	0.69	1.198	0.519	0.330	0.735	1.065	22.8	0.66	0.9939	
	40	0.81	1.601	0.909	0.371	0.688	1.059	27.1	0.79	0.9971	

**Table 3:** TGA data for the pEGF45 samples cured at different temperatures.

Sample	Curing Temperature T, °C	Temperature Range T, °C		Residue, %
		I Stage	II Stage	
pEGF45	20	170~360	361~530	14.5
	30	175~363	364~544	14.7
	40	177~365	366~550	15.1

**Table 4:** Designations and formulations of the investigated samples.

Sample	p-EGF, mol.%	AA, mol.%	BPO, %	DMA, %
pEGF30	30.3	69.7	1	0.15
pEGF45	45.4	54.6	1	0.15
pEGF60	60.5	39.5	1	0.15

## Data Availability

The original contributions presented in this study are included in the article. Further inquiries can be directed to the corresponding author(s).

## Supplementary Materials

The following supporting information can be downloaded at https://www.mdpi.com/article/10.3390/molecules30194020/s1. Figure S1: Time-dependent graphs of storage modulus and loss modulus during the isothermal curing pro-cess of pEGF30 system; Figure S2: Time-dependent graphs of storage modulus and loss modulus during the isothermal curing pro-cess of pEGF60 system.

## Abbreviations

The following abbreviations are used in this manuscript:

AA	acrylic acid
BPO	benzoyl peroxide
DSC	differential scanning calorimetry
DMA	dimethyl aniline
LVR	linear viscoelastic region
PCM	polymer composite materials
pEGF	Poly(ethylene glycol fumarate)
TGA	thermogravimetric analysis
UP	unsaturated polyester
UPR	unsaturated polyester resins
