# Supplementary material for: Thermosetting Resins Based on Poly(Ethylene Glycol Fumarate) and Acrylic Acid: Rheological and Thermal Analysis
Source: Molecules. 2025 Oct 8;30(19):4020. doi: 10.3390/molecules30194020 (PMC12526166; doi:10.3390/molecules30194020)
Supplement: Supplementary file 1 [file molecules-30-04020-s001.zip › molecules-3884767-supplementary.pdf]

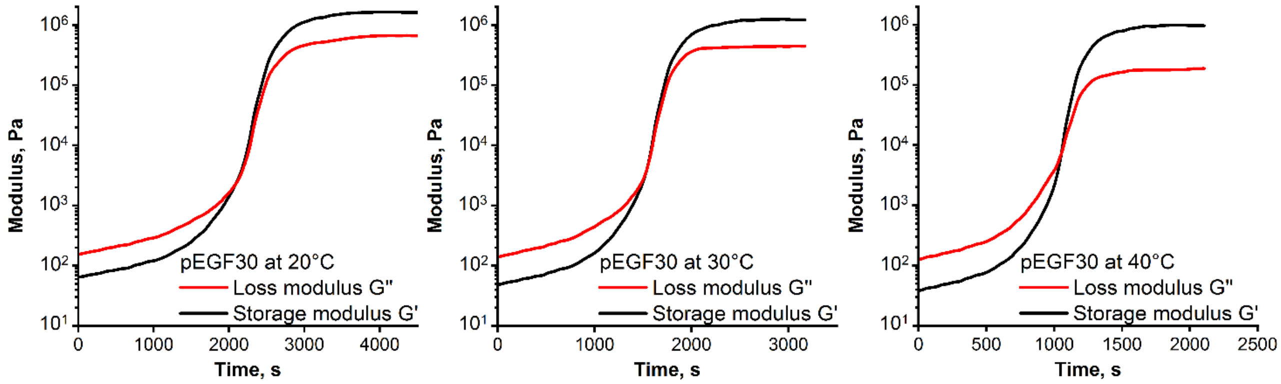

Figure S1. Time-dependent graphs of storage modulus and loss modulus during the isothermal curing process of pEGF30 system.

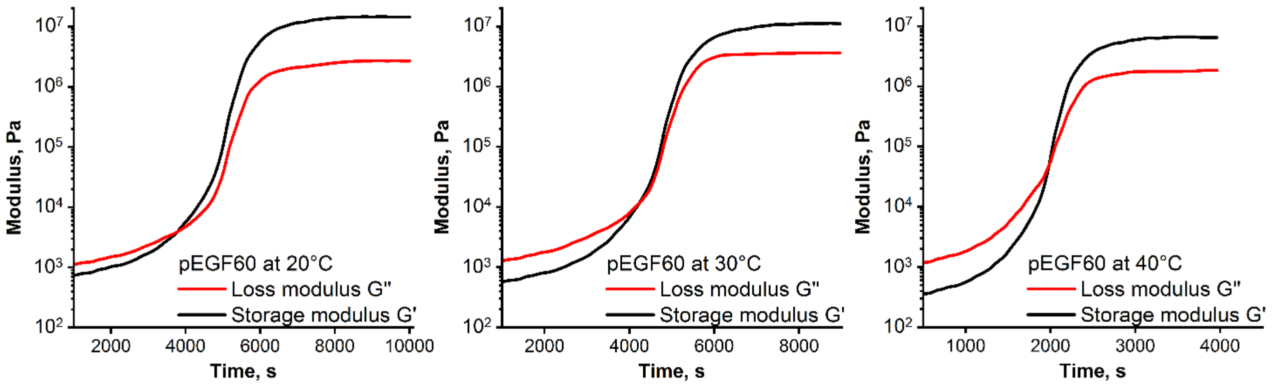

Figure S2. Time-dependent graphs of storage modulus and loss modulus during the isothermal curing process of pEGF60 system.
